# Undifferentiated carcinoma of the pancreas with osteoclast-like giant cells: a case report

**DOI:** 10.1186/s13256-023-04213-4

**Published:** 2023-11-16

**Authors:** William Chan, Sungmee Park, Layla Shirkhoda, Ryan O’Connell, Roozbeh Houshyar

**Affiliations:** 1 Irvine Department of Radiological Sciences, University of California, 101 The City Drive South, Orange, CA 92868 USA; 2 Irvine Department of Pathology and Laboratory Medicine, School of Medicine, University of California, University of California Irvine, Irvine, CA 92697 USA

**Keywords:** Undifferentiated carcinomas of the pancreas with osteoclast-like giant cells, Pancreatic neoplasm, Case series

## Abstract

**Background:**

Undifferentiated carcinomas of the pancreas with osteoclast-like giant cells (UCPOGC) are rare pancreatic neoplasms that account for less than 1% of all pancreatic malignancies. This case report of a 54-year-old male with metastatic UCPOGC adds to the existing literature and further ascertains the clinical and imaging features, treatment options, and prognosis of this rare entity.

**Case presentation:**

We present the detailed clinical course of a 54-year-old Asian male patient with UCPOGC, with focus on the relevant clinical features and imaging findings that are characteristic of this disease entity.

**Conclusions:**

UCPOGC is an extremely rare pancreatic tumor with a unique histopathology and clinical course. It is often difficult to distinguish UCPOGCs from other pancreatic tumors, such as traditional pancreatic ductal adenocarcinomas (PDAC), on imaging, and it therefore remains a pathological diagnosis. Surgery is generally regarded as the first-line treatment option, and the roles of chemotherapy and radiation are unclear. Due to the exceeding rarity of this tumor, large-scale clinical studies are not feasible. Therefore, it is important to share individual insights and experiences to improve our understanding and care for patients with this devastating disease.

## Background

Undifferentiated carcinomas of the pancreas with osteoclast-like giant cells (UCPOGC) are rare pancreatic neoplasms that account for less than 1% of all pancreatic malignancies [1]. UCPOGCs are thought to originate from ductal epithelium [1–3], and they are composed of two distinct cell populations: neoplastic, pleomorphic, mononuclear cells and benign, osteoclast-like giant cells (OGC), which are thought to be secondarily recruited into the tumor [1, 4–7]. The prognosis is generally poor [1], but literature suggests that UCPOGCs may have a more favorable prognosis and lower recurrence rate compared to traditional pancreatic ductal adenocarcinomas (PDAC) and other subtypes of undifferentiated carcinomas of the pancreas [1, 8, 9].

Currently, literature on UCPOGCs is lacking relative to other pancreatic malignancies, and our knowledge of UCPOGCs is largely based on case reports. Therefore, it is important to continue adding to current knowledge to further ascertain the clinical and imaging features, treatment options, and prognosis for this rare entity. Here, we present a case of metastatic UCPOGC in a 54-year-old male.

## Case presentation

A 54-year-old Asian male patient with a history of hypertension, pancreatic cysts, colonic tubular adenomas, and family history of colonic polyps presented for evaluation of a large peri-splenic and pancreatic mass that was discovered incidentally on endoscopic ultrasound (EUS). The patient had negative genetic testing for MUTYH-associated polyposis and familial adenomatous polyposis. The patient had experienced 30 pounds of weight loss over the past year, but he reported this was due to intentional efforts to lose weight. He was otherwise asymptomatic without abdominal pain, nausea, vomiting, hematemesis, hematochezia, melena, or jaundice.

The patient’s abdominal physical examination was unremarkable without palpable mass. Laboratory examination revealed mild anemia with a hemoglobin of 11.7 g/dL (normal < 13.5 g/dL). Carbohydrate antigen (CA) 19–9 was 138 U/mL (normal < 35 U/mL), and carcinoembryonic antigen (CEA) was 2.4 ng/mL (normal < 3.0 ng/mL). Repeat EUS completed 2 months after initial EUS revealed a 12 cm, isoechoic to hyperechoic, heterogenous, cystic splenic mass. There was no abscess, mucinous, or solid component. Fine-needle aspiration of the cyst revealed serosanguinous, non-mucinous (negative string sign), and non-purulent material. The fluid cultures including an acid-fast bacilli culture were negative. The fluid CEA level was 2.6 ng/mL (normal 0.0—3.8 ng/mL).

**Fig. 1:**
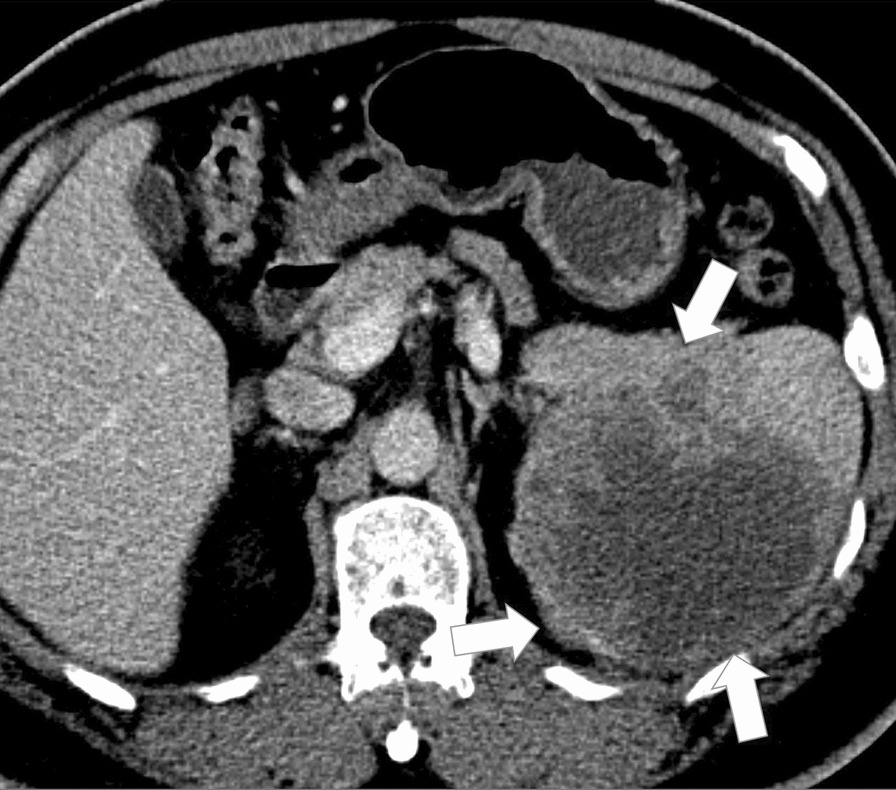
Contrast-enhanced abdominal CT showing a large heterogeneous and hypodense mass (arrows) involving the pancreatic tail and spleen

**Fig. 2:**
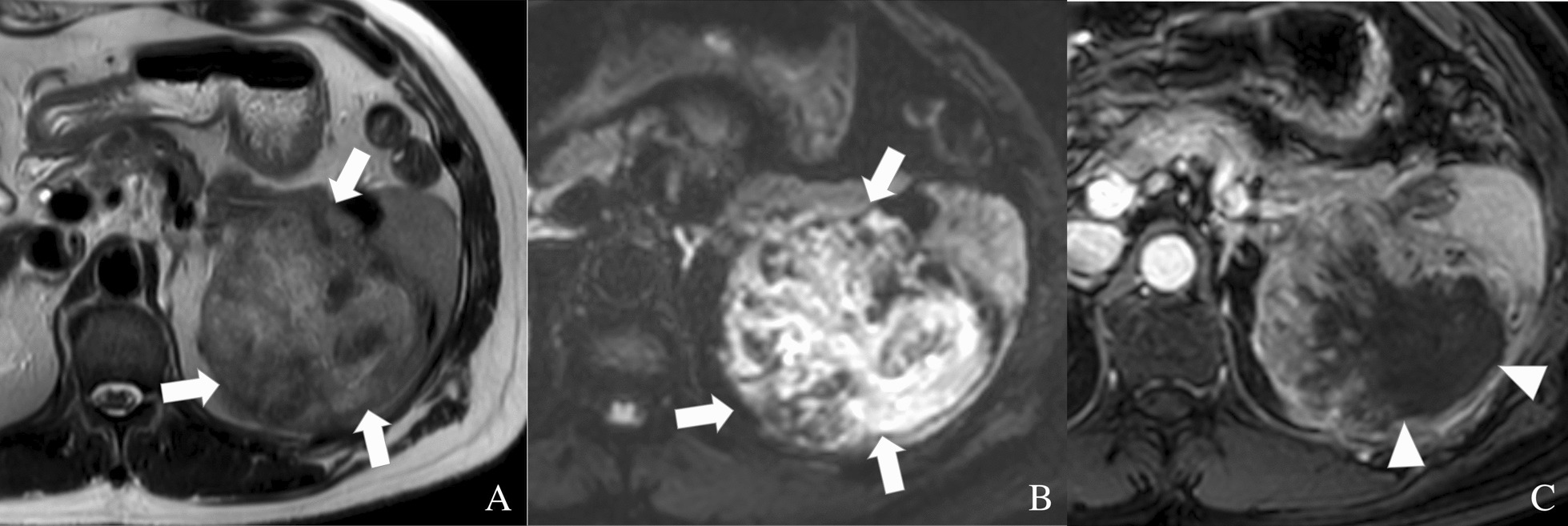
Abdominal MRI showing a large mass with heterogenous intermediate T2-signal (arrows) (**A**), heterogenous restricted diffusion (arrows) (**B**), and heterogenous peripheral enhancement on T1 fat sat post-contrast (arrowheads) imaging (**C**) involving the pancreatic tail and spleen. Overall findings demonstrate a relatively circumscribed, large mass with solid and fluid components and peripheral solid enhancement suggestive of high cellularity and central necrosis invading the splenic parenchyma

Contrast-enhanced abdominal computed tomography (CT) demonstrated a large, heterogeneous, hypodense mass involving the pancreatic tail and spleen (Fig. 1). Abdominal MRI showed a large mass with heterogenous intermediate T2-signal (Fig. 2A), heterogenous restricted diffusion (Fig. 2B), and heterogenous peripheral enhancement on T1 fat sat post-contrast imaging (Fig. 2C) involving the pancreatic tail and spleen. Overall findings demonstrate a relatively circumscribed, large mass with solid and fluid components and peripheral solid enhancement suggestive of high cellularity and central necrosis, invading the splenic parenchyma. No lymphadenopathy was noted.

**Fig. 3:**
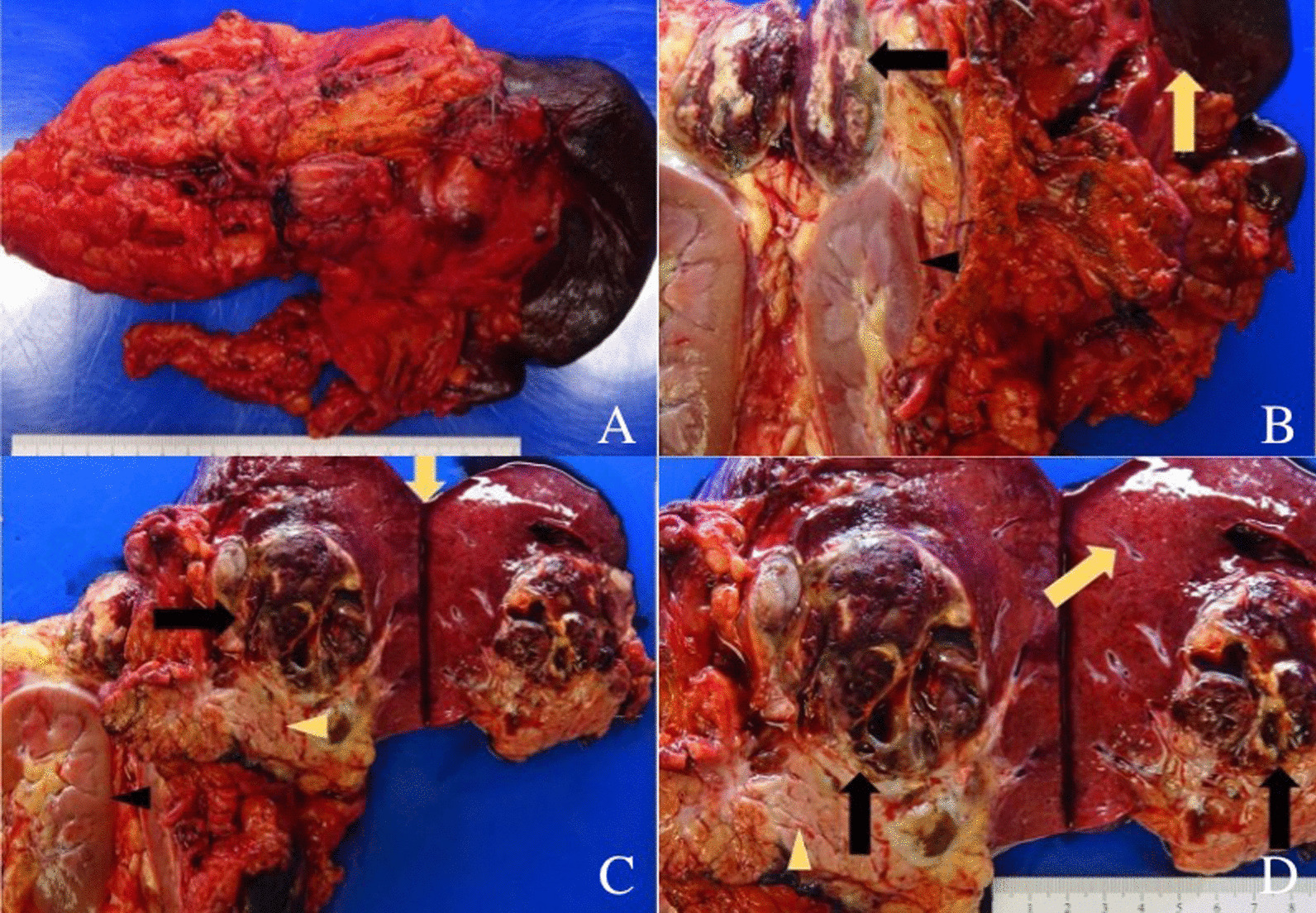
Intact gross specimen (**A**). Gross photograph showing left kidney (black arrowhead) and tumor (black arrow) sectioned in addition to the intact spleen (yellow arrow) (**B**). The tumor is seen extending to the superior region of the left kidney abutting the renal capsule; hemorrhagic and necrotic changes are present centrally within the tumor (**B**). Gross photograph showing the pancreas (yellow arrowhead), tumor (black arrow), left kidney (black arrowhead), and spleen (yellow arrow) sectioned (**C**). Gross photograph showing the tumor (black arrow), spleen (yellow arrow), and pancreas (yellow arrowhead) sectioned; Here, the tumor is seen extending from the pancreas to the spleen with hemorrhagic and cystic spaces (**D**)

**Fig. 4:**
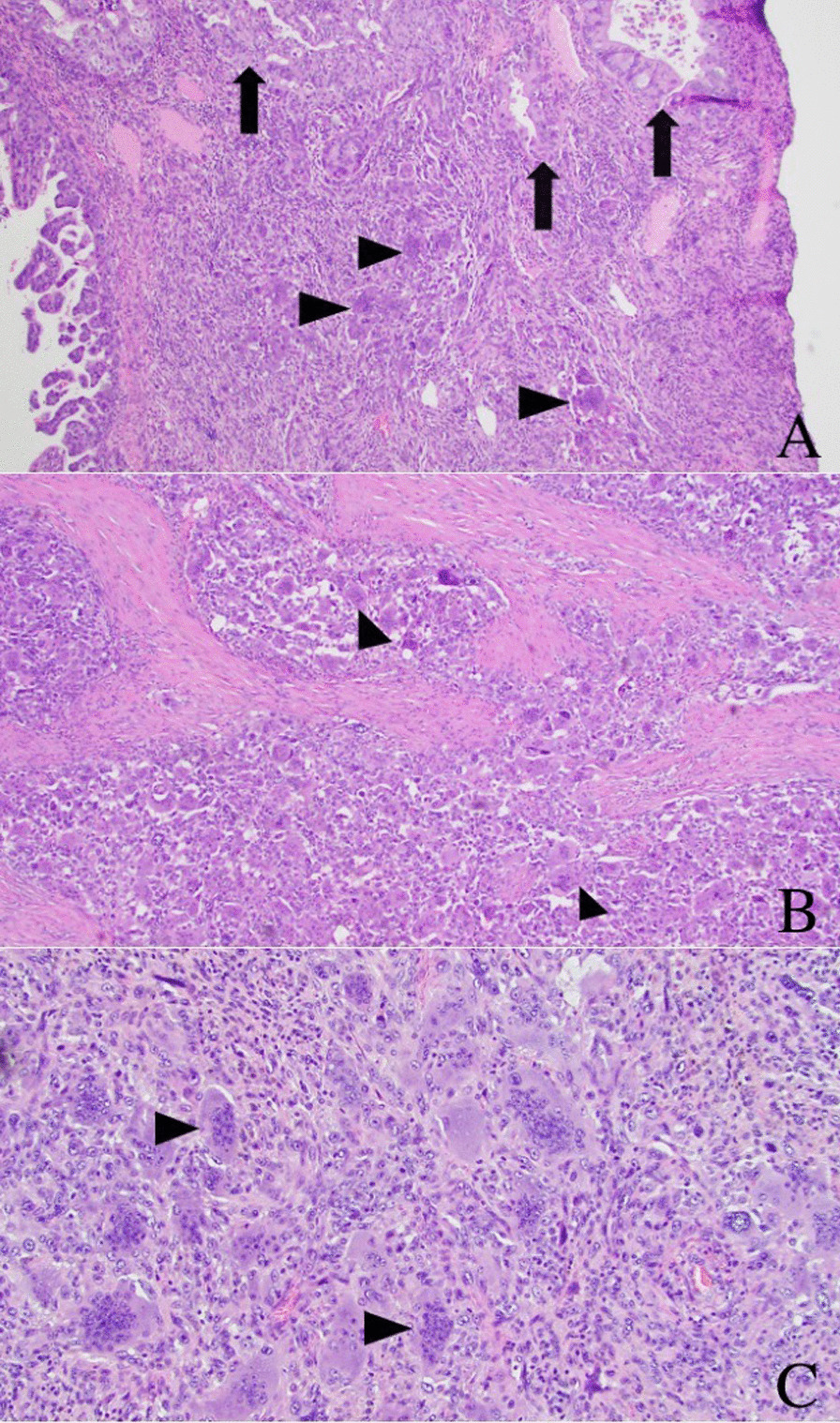
40 × magnification image showing regions of more conventional adenocarcinoma morphology (arrows) with multiple giant cells seen centrally within the image (arrowheads) (**A**). 40 × magnification image showing one of the large regions of the tumor that contained predominantly giant cells (arrowheads) (**B**). 100 × high magnification image showing the osteoclast-like morphology of the giant cells (arrowheads) (**C**)

**Fig. 5:**
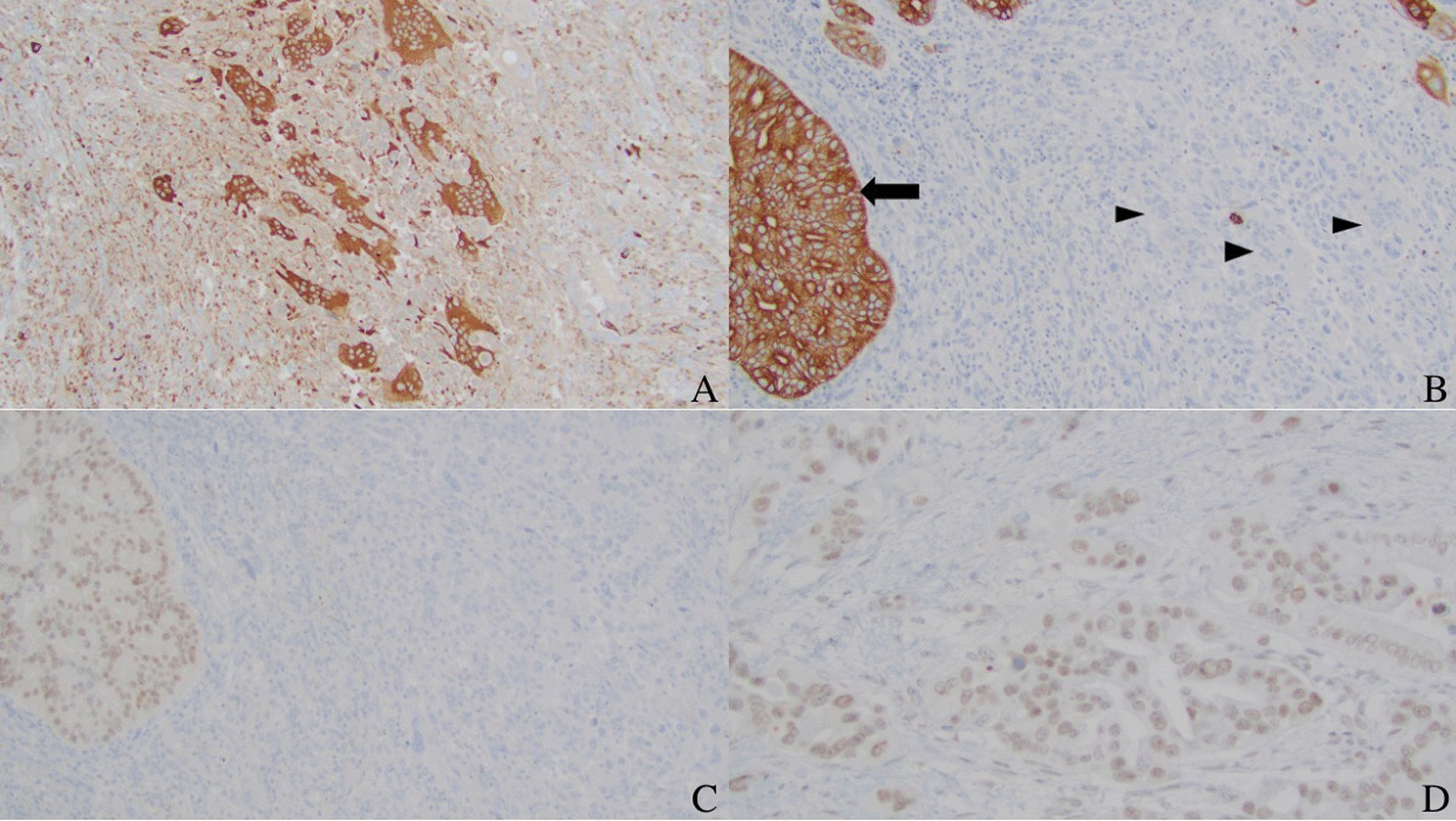
100 × magnification image showing positive CD68 staining in giant cells (**A**). 100 × magnification image showing cytokeratin (AE1/AE3) positivity within the adenocarcinomatous component (arrow), and negative within giant cells (arrowheads) (**B**). 100 × magnification image showing positive CDX2 staining in traditional adenocarcinoma component and negative in giant cells (**C**). 200 × magnification image showing positive INI-1 staining within the conventional adenocarcinoma component (**D**)

An en-bloc open distal pancreatectomy, splenectomy, left nephrectomy, left adrenalectomy, partial gastrectomy, and diaphragm resection were performed to remove the mass. The mass grossly involved both the distal pancreas and spleen but not the left adrenal gland, left kidney, or stomach. Gross inspection revealed a 11.0 × 8.1 × 7.0 cm tan-yellow to red-brown tumor that extended from the pancreas to the spleen and superior aspect of the left kidney (Fig. 3A–D). Further sectioning revealed hemorrhagic and necrotic-appearing cut surfaces with cystic spaces. Microscopically, multinucleated OGCs were present with areas of traditional adenocarcinomatous morphology (Fig. 4A–C). On immunohistochemistry, the OGCs were positive for CD68 and negative for cytokeratin (AE1/AE3) and CDX2 (Fig. 5A–C). The adenocarcinoma component was positive for AE1/AE3, CDX2, and INI-1 (Fig. 5B–D). The final diagnosis was UCPOGC of the pancreatic tail with direct tumor extension into the spleen.

The patient had no evidence of recurrent disease on six-week post-op CT, and he was started on six months of adjuvant modified FOLFIRINOX (mFFX) at nine weeks post-op. Unfortunately, on repeat CT scan 20 weeks post-op, a 1.2 cm right hepatic lobe liver lesion was discovered, and the biopsy was consistent with metastasis from the primary pancreatic UCPOGC. The mFFX was stopped and the patient was started on gemcitabine plus nab-paclitaxel. The patient completed two cycles of gemcitabine plus nab-paclitaxel before unfortunately expiring 11 months post-op.

## Discussion and conclusions

UCPOGC is a rare pancreatic tumor that may arise anywhere along the pancreas and typically presents in the fifth to seventh decades of life with symptoms such as abdominal or back pain, palpable mass, weight loss, and/or jaundice [1, 4–7, 10]. Upon presentation, our patient only experienced weight loss, but it was unclear whether this was purely intentional or secondary to the tumor. Asymptomatic cases have been reported, though UCPOGCs in asymptomatic cases tend to be smaller in comparison [11–13]. At their time of discovery, UCPOGCs are generally quite large, measuring an average of 8 cm, which may be due to their rapid growth [14, 15]. Our specimen was 11.0 × 8.1 × 7.0 cm which is relatively large [1, 6, 7], so it is surprising that our patient had no bulk or obstructive symptoms. In contrast, a 57-year-old male presenting with a nearly identical tumor size and location experienced epigastric pain and weight loss [14].

Involvement of the spleen in cases of UCPOGC is well-described [1, 4, 5]. Our specimen analysis revealed the primary site in the distal pancreas, with the splenic involvement because of local invasion by the primary pancreatic UCPOGC, a previously described phenomenon [14].

Our patient had an elevated CA 19-9 of 138 U/mL (normal < 35 U/mL) but normal serum CEA of 2.4 ng/mL (normal < 3.0 ng/mL) and fluid CEA of 2.6 ng/mL (normal 0.0–3.8 ng/mL). In previous cases of UCPOGCs, both normal and elevated levels of CA 19-9 and CEA have been described [1, 4, 5, 9, 11, 16, 17].

The sensitivity of CT, MRI, and EUS for the detection of UCPOGC is similar to that for the detection of traditional PDAC [5]. The specificity of these imaging modalities remains in question, though there are some features that may help distinguish UCPOGC from PDAC. On CT, UCPOGCs are described as heterogenous masses with cystic or solid features [1, 4–7, 10, 16]. Contrast enhancement may or may not be present. In this case, contrast-enhanced CT revealed a heterogeneous and hypodense mass. This mass features thick solid peripheral contrast enhancement, with suggestion of central necrosis. CT features that may help differentiate UCPOGC from PDAC include the presence of contrast enhancement and regularity of the tumor border. PDACs are typically hypoattenuating tumors with ill-defined borders, whereas UCPOGCs are well-defined and contrast enhancement is not uncommon [16, 18]. The hyperemic nature of UCPOGCs is expected given the rapid growth of UCPOGCs [14].

In our case, abdominal MRI of the UCPOGC demonstrated heterogenous intermediate T2-signal, marked restricted diffusion, and heterogenous peripheral T1 fat saturated post-contrast enhancement. In the literature, UCPOGCs have a variable appearance on MRI, with no consistent signal intensity described for each MRI sequence. UCPOGCs have been described as hypo- or hyperintense on T1, T2, and diffusion-weighted imaging [4, 10, 14]. Isointense UCPOGCs on T2 and diffusion-weighted sequences have also been described [10]. In contrast, PDACs exhibit low signal intensity on T1-weighted imaging, but also appear variable on T2 and diffusion-weighted sequences [18]. In our study, EUS of the UCPOGC revealed a heterogenous, isoechoic to hyperechoic mass. The heterogenous echotexture of UCPOGCs on EUS is a well-described feature [19–21], and its presence may help distinguish UCPOGCs from PDACs, which are more uniformly hypoechoic [18].

UCPOGCs are characterized by pleomorphic mononuclear neoplastic cells with intermixed, benign osteoclast-like giant cells (OGC), which are the histopathologic hallmark of UCPOGCs. In our case, the OGCs were CD68 positive and cytokeratin AE1/AE3 negative. This immunohistochemical profile is consistent with previously reported cases of UCPOGC and reinforces the idea that OGCs are derived from a benign histiocytic cell population [1, 11, 14, 20, 22]. OGCs are thought to be secondarily recruited into the tumor via chemotaxis [3], and their presence is associated with an improved prognosis compared to PDACs and undifferentiated carcinomas of the pancreas without OGCs [1, 8, 9].

Muraki *et al.* reported a 5-year survival of 59.1% and median survival of 7.67 years for patients with UCPOGC, versus 15.7% 5-year survival and 1.59-year median survival for PDACs [1]. As seen in our patient, UCPOGCs are associated with concomitant PDAC with composite tumors in up to 76% of cases [1], which results in a worse prognosis [8, 9]. Luchini *et al.* reported a median 15-month survival in a group of 9 patients with UCPOGC with associated PDAC, and our patient exhibited 11-month survival post-op [8]. In our case, the PDAC component stained positive for CDX2, which is expressed in normal pancreatic ductal epithelium and appears to be downregulated during the transition to PDAC [23]. Its expression in PDAC may indicate a worse prognosis [23], although it is unclear if a CDX2-positive PDAC component in UCPOGC is associated with worse outcomes. The UCPOGC in this case also stained positive for INI-1, which is consistent with Reid *et al.* study [7], where three out of three UCPOGCs retained INI-1 stain. INI-1 staining may be a helpful distinguishing feature, as Agaimy *et al.* demonstrated loss of INI-1 in all four monomorphic anaplastic subtypes of pancreatic undifferentiated rhabdoid carcinoma [24].

The optimal treatment for UCPOGCs remains unstandardized. Surgery is considered first-line treatment [25], and in our case, the UCPOGC was removed via en-bloc resection. The roles of chemotherapy and radiation are unclear. There are case reports of successful treatment with adjuvant chemotherapy, with one patient recurrence-free 10 years after en-bloc resection of UCPOGC with adjuvant gemcitabine [5]. However, UCPOGCs unresponsive to adjuvant chemotherapy have also been described [26]. In our case, the patient was treated with adjuvant mFFX based on the presence of concurrent PDAC but developed liver metastasis while on treatment. As a result, he was switched to gemcitabine plus nab-paclitaxel which has demonstrated efficacy in undifferentiated pancreatic carcinomas [27], however the patient unfortunately expired after two cycles. Adjuvant radiation has theoretical benefit given the radiosensitivity of giant cell tumors of bone, although its effectiveness has not been fully established [22]. There is a paucity of data regarding therapy for metastatic UCPOGC, with some reports of benefit with pembrolizumab [28, 29]. Interestingly, expression of programmed death-ligand 1 is a negative prognostic factor for UCPOGCs [30], which may explain the efficacy of pembrolizumab in metastatic disease.

In summary, UCPOGCs are extremely rare pancreatic malignancies with a unique histopathology and clinical course. Certain radiologic findings may be helpful to distinguish UCPOGC from PDAC, but they are generally non-specific and UCPOGC remains a pathological diagnosis. If possible, surgery is generally accepted as the first-line treatment option, whereas the roles of chemotherapy and radiation are unclear, and their evidence is primarily anecdotal. Due to the exceeding rarity of this tumor, large-scale clinical studies are unlikely to be feasible, therefore contributing individual insights and experiences will be important in promoting better understanding and care for patients with this devastating disease.

## Data Availability

Not applicable.

## Abbreviations

UCPOGC: Undifferentiated carcinoma of the pancreas with osteoclast-like giant cells
PDAC: Pancreatic ductal adenocarcinoma
OGC: Osteoclast-like giant cells
EUS: Endoscopic ultrasound
CA: Carbohydrate antigen
CEA: Carcinoembryonic antigen
CT: Computed tomography
MRI: Magnetic resonance imaging
T2-signal: T2-weighted signal
T1 fat sat: T1-weighted fat-saturated
CD68: Cluster of differentiation 68
AE1/AE3: Cytokeratin markers
CDX2: Caudal-type homeobox 2
INI-1: Integrase interactor 1
PD-L1: Programmed death-ligand 1
mFFX: Modified FOLFIRINOX
nab-paclitaxel: Nanoparticle albumin-bound paclitaxel
